# Overexpression and alternative splicing of NF-YA in breast cancer

**DOI:** 10.1038/s41598-019-49297-5

**Published:** 2019-09-10

**Authors:** Diletta Dolfini, Valentina Andrioletti, Roberto Mantovani

**Affiliations:** 1 Dipartimento di Bioscienze, Università degli Studi di Milano, Via Celoria 26, 20133 Milano, Italy; 2 Present Address: Internal Medicine VIII, University Hospital Tübingen, Otfried-Müller-Str. 14, 72076 Tübingen, Germany

**Keywords:** Breast cancer, Cancer genomics

## Abstract

NF-Y is a CCAAT-binding trimeric transcription factor, whose regulome, interactome and oncogenic potential point to direct involvement in cellular transformation. Yet little is known about the levels of NF-Y subunits in tumors. We focused on breast carcinomas, and analyzed RNA-Seq datasets of TCGA and 54 BRCA cell lines at gene and isoforms level. We partitioned all tumors in the four major subclasses. NF-YA, but not histone-fold subunits NF-YB/NF-YC, is globally overexpressed, correlating with the proliferative Ki67 marker and a common set of 840 genes, with *cell-cycle*, *metabolism* GO terms. Their promoters are enriched in NF-Y, GC-rich and E2F sites. Surprisingly, there is an isoform switch, with the “short” isoform -NF-YAs- becoming predominant in tumors. E2F genes are also overexpressed in BRCA, but no switch in isoforms is observed. In Basal-like Claudin^low^ cell lines and tumors, expression of NF-YAl -long- isoform is high, together with 11 typical EMT markers and low levels of basal Keratins. Analysis of Progression-Free-Intervals indicates that tumors with unbalance of NF-YA isoforms ratios have worst clinical outcomes. The data suggest that NF-YA overexpression increases CCAAT-dependent, pro-growth genes in BRCA. NF-YAs is associated with a proliferative signature, but high levels of NF-YAl signal loss of epithelial features, EMT and acquisition of a more aggressive behavior in a subset of Claudin^low^ Basal-like tumors.

## Introduction

The synergy and precise interplay of Transcription Factors -TFs- on promoters and enhancers dictate regulation of gene expression. Many TFs are pivotal in the control of cell growth, and their altered structure or expression leads to tumorigenesis. NF-Y is a TF binding with high specificity to the CCAAT box, an important regulatory element. NF-Y has a role as a”pioneer” TF, setting the chromatin stage for recruiting other TFs and coactivators^1–3^. It consists of three subunits: the histone fold domain -HFD- dimer NF-YB/NF-YC and the sequence-specific NF-YA^4^. NF-YA and NF-YC are involved in alternative splicing^5,6^. Specifically, there are two major isoforms of NF-YA, NF-YAs “short” and NF-YAl “long”, differing in 28/29 amino acids within the Gln-rich TransActivation Domain, TAD^5^.

NF-Y genes are rarely mutated in cell lines or cancer specimens (http://www.sanger.ac.uk/genetics/CGP/cosmic/), yet different lines of evidence suggest that it plays a relevant role in cancer progression. Microarrays profiling of genes overexpressed in tumor vs normal cells found cancer “signature” genes and TFBSs -Transcription Factor Binding Sites- searches identified CCAAT as overrepresented in their promoters (Reviewed in Ref.^7^). The same conclusion was reached in Oncomine profiling data using unbiased *de novo* motif discovery tools^8^. More recent profiling reports confirmed this, specifically in breast cancer^9–12^. RNA-seq data analysis are fewer, but pointing in the same direction^13,14^. It is well established that CCAAT, wherever present in promoters, is crucial for high-level expression of genes^15^; thus, it appears that tumors rely on CCAAT-binding to activate a significant number of “cancer“ genes. NF-Y was analyzed by the vast ENCODE consortium, and by independent ChIP-Seq experiments: connections to oncogenic and growth controlling TFs and signaling pathways emerged (1, Reviewed by^16^).

What is not clear is whether NF-Y is overexpressed in cancer cells, and in case, which types. There is no widespread, systematic analysis of expression levels of the subunits in tumors, and the available information is limited to small cohorts of specific cancers. Epithelial ovarian cancer cells show increased NF-YA levels, specifically the short isoform, and tumors with high NF-YA levels have a poorer prognosis^17,18^. Elevated expression of NF-YA, along with other TFs, was reported in Triple Negative Breast Cancers^14^. High levels of NF-YA mRNA were found in the “diffuse” type of gastric cancer^19^, and of the NF-YC protein in gliomas^20^ and colon adenocarcinomas^21^.

To close this gap in our knowledge of NF-Y biology, we analyzed the mRNA levels of NF-Y subunits in human tumor samples, both in quantitative and qualitative terms, by interrogating large-scale RNA-Seq datasets of TCGA. We then decided to focus specifically on breast carcinomas.

## Results

### NF-YA is widely overexpressed in tumors of epithelial origin

The global mRNA levels of the three NF-Y subunits were investigated with Firebrowse (http://firebrowse.org/viewGene.html) in 37 different types of tumors present in TCGA. 9 types of tumors lack normal counterparts, and were not further considered. We restricted analysis to tumors with matched normal samples >5. Therefore, the analysis was limited to 18 tumor types and the results are shown in Fig. S1 as FPKMs box plots of NF-YA, NF-YB and NF-YC. The levels of NF-YA are increased in many types of tumors and decreased in few. Considering a p-value threshold of e-04, 11/18 tumors have higher levels of NF-YA, 2/18 lower levels. The increase is robust in epithelial tumors: carcinomas of breast (BRCA), colon (COAD), rectum (READ), stomach (STAD), liver (LIHC), prostate (PRAD), uterine (UCEC), head and neck squamous cells (HNCC), cholangiocarcinoma (CHOL), lung adenocarcinoma (LUAD) and squamous cells carcinoma (LUSC). The pattern is different for the HFD subunits, since overexpression is neither statistically overwhelming nor concordant: NF-YB is decreased in 7 tumors, increased in 5; NF-YC is increased in 6 and decreased in 3. An increase in all NF-Y subunits is observed in CHOL, LIHC (Liver hepatocellular carcinoma) and STAD, a decrease in THCA (thyroid carcinoma) and KICH (kidney chromophoebe). In ESCA (Esophageal carcinoma), KIRP (kidney renal papillary cell carcinoma) and GBM (glioblastoma multiforme), subunits expression is not changed. In conclusion, there is an increase in mRNA levels of NF-YA, but not NF-YB/NF-YC, in most tumors, specifically of epithelial origin.

One of the tumors in which overexpression of NF-YA is not observed is GBM. To verify this, we searched independent RNA-seq GEO datasets (GSE59612) that include samples taken from areas of tumors with mesenchymal and neural cells and matched with normal ones^22^. Box plot analysis of expression of the two major splicing isoforms of NF-YA did not show a significant change; the same was true for the three isoforms of NF-YC, bar a modest increase in the 37 kD and a decrease of the 50 kD isoform. NF-YB was decreased (Fig. S2). These results confirm the TCGA data shown above in that there is no overexpression of NF-YA in GBM.

### NF-YA is overexpressed in BRCA

**Figure 1:**
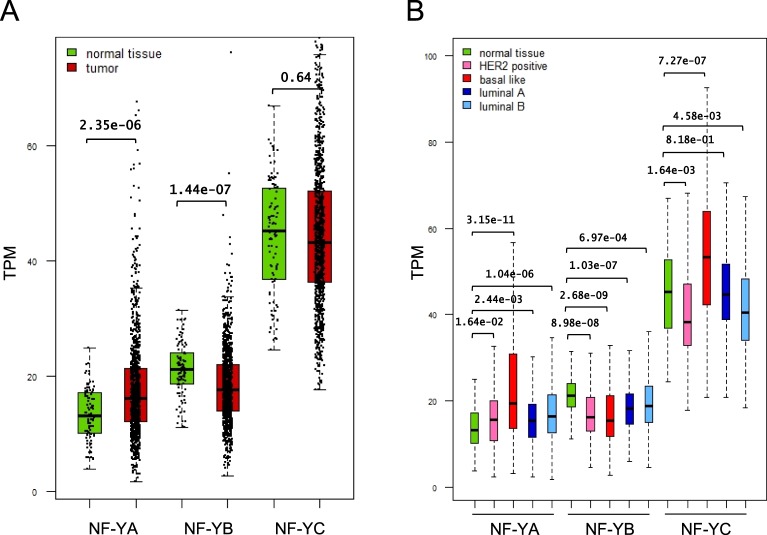
Analysis of NF-Y subunits expression in TCGA BRCA. (**A**) Box plots of NF-Y subunits expression at gene level in TCGA-BRCA, measured in TPMs. (**B**) Expression of NF-Y subunits at gene level across the TCGA BRCA subtypes after PAM50 classification of TCGA-BRCA cohort. p-values are calculated using a Wilcoxon signed-rank test.

We focused our attention on the BRCA dataset of TCGA: further quantitative analysis of RNA-Seq data found that the levels of NF-YA, but not NF-YB nor NF-YC, are increased in cancer samples compared to normal controls (Fig. 1A). Breast carcinomas are divided in several subtypes, according to different clinical, histological and molecular parameters. In theory, NF-YA overexpression could be specific to one -or more- of the cancer subtypes. Molecular classification of BRCA is defined by a gene expression signature of 50 genes -termed PAM50- partitioning four types: Basal-like, HER2E, Luminal A and Luminal B. Originally identified with mRNA profilings^23^, PAM50 was later confirmed by qRT-PCR^24^, RNA-Seq and partial analysis of TCGA samples^25^. Classification of the four subtypes within TCGA was performed on 514^26^ and later 817 tumor samples^27^. Our first goal was to extend it to all 1083 BRCA for which RNA-Seq data are available. To do so, we employed a classifier based on PAM50, as defined previously^28^. Venn diagrams of the 514, 817 and 1083 samples are shown in Fig. S3: with respect to the original partitioning of 524 tumors^26^, relative proportions are very similar for Basal-like (now 203 tumors) and HER2E (now 126); we confirm a shift of samples from Luminal A -now 320 tumors- to Luminal B -now 425- as previously described^27^. The expression heatmap of PAM50 genes in all BRCA samples shows the expected clustering (Fig. S4). Supplementary Table 1 shows the complete list of 1083 BRCA tumors classified according to the four subtypes. With this in hand, we compared the mRNA levels of the three subunits in the four subtypes to the 113 normal breast samples: Fig. 1B shows global increase of NF-YA in all subtypes, somewhat less significant in HER2E, and very significant in Basal-like (p value e-11). NF-YB is not affected, bar a statistically significant decrease in Basal-like. NF-YC shows some reduction in HER2E and modest increase in Basal-like. These data indicate that overexpression of NF-YA is not restricted to a specific subtype of BRCA, and confirm little to no change in HFD subunits.

### NF-Y, GC-rich and E2F sites are enriched in promoters of common genes overexpressed in all BRCA subtypes

**Figure 2:**
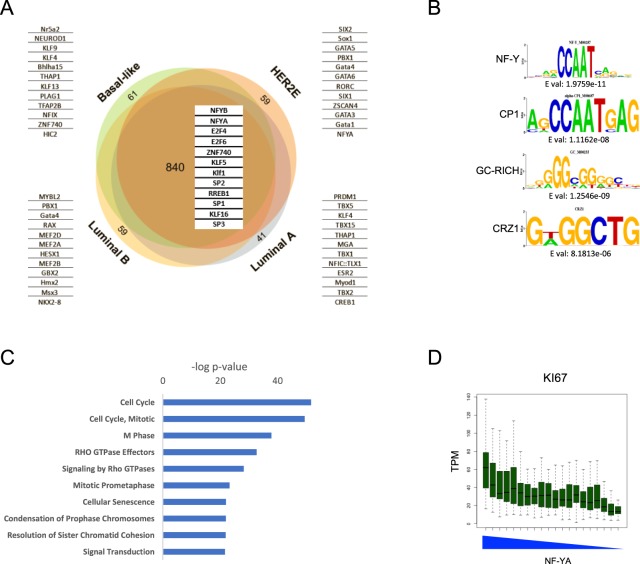
Analysis of gene expression in TCGA BRCA. (**A)** Venn diagrams show the upregulated genes for each PAM50 subtype, comparing subtype samples to normal tissues in the TCGA BRCA cohort. On the borders, genes exclusively upregulated in each subtype are shown. For subtype-specific and common upregulated genes, the most represented promoter TFBSs are listed, obtained using the Pscan software. (**B**) The most represented motifs in the commonly upregulated genes from *de novo* discovery using Weeder. (**C**) The most represented Reactome pathways enriched in commonly upregulated genes are listed according to their p-value. The list is obtained using KOBAS. (**D**) Expression of levels of the proliferative marker Ki67 across TCGA-BRCA tumor samples ranked based on NF-YA expression.

We analyzed the levels of gene expression in the four BRCA subtypes (Supplementary Table 2). The lists of the selected over- and under-expressed genes, using Log_2_FC 2 and FDR 0,01 thresholds, are in Supplementary Table 3. The overlap between BRCA subtypes is quite extended, with some 840 genes commonly overexpressed, while 41–61 genes are specific for each subtype (Fig. 2A). A similar picture was observed in down-regulated genes (Fig. S5). We then analyzed promoter sequences -from −450 to +50 from the TSS- of overexpressed genes with the Pscan tool^29^: this algorithm allows the retrieval of statistically enriched TFBSs (Transcription Factors Binding Sites) based on the DNA matrices present in the JASPAR database. The results are shown in Fig. 2A: each of the four distinct signatures contains a specific set of promoters TFBSs, different from each other. In the commonly up-regulated genes, the most enriched matrices are CCAAT/NF-Y, flavors of E2Fs (E2F4/6) and GC-rich matrices, binding to Zinc-finger TFs (KLFs, SP1/2/3). NF-Y or E2Fs sites are not found in the subtypes-specific up-regulated genes. The same analysis was performed on promoters of down-regulated genes, and neither NF-Y nor E2F were found (Fig. S5). To verify these results, we run Weeder, an algorithm for *de novo* motif discovery finding matrices without any pre-existing bias^30^. We found the NF-Y/CCAAT matrix with high frequency, in addition to a GC-rich matrix (Fig. 2B). E2F was not identified with this method. In summary, it can be concluded that NF-Y/CCAAT is a centerpiece in promoters of 840 genes commonly overexpressed in TCGA breast carcinomas.

We then analyzed the common and subtype-specific overexpressed genes for Gene Ontology terms using the KOBAS algorithm: the most enriched term in the commonly overexpressed genes is *cell-cycle*, specifically *mitosis*. Additional enriched terms are *signaling* and *senescence*. On the other hand, pathways-specific terms are distinctly enriched in genes overexpressed in the four subtypes of BRCA (Fig. 2C). These results suggest that the presence of the NF-Y-binding CCAAT matrix correlates with a signature of overexpressed “proliferative” genes. To confirm this, we ranked all BRCA tumors in 20 groups, according to NF-YA levels (Fig. 2D, Lower Panel) and observed the levels of Ki67, a proliferative marker: Ki67 is indeed progressively decreased from NF-YA^high^ to NF-YA^low^ groups (Fig. 2D, Upper Panel). Incidentally, Ki67 is a direct genomic target of NF-Y (Not shown). In conclusion, increased NF-YA levels positively correlates with a “proliferative” signature of genes containing CCAAT in promoters, and with a marker of proliferation.

### E2Fs overexpression in BRCA

The discovery of the E2F matrix in the BRCA commonly overexpressed genes was not a surprise, as it is often found in tumor cohorts^8^. E2Fs are a family of 8 genes and microarrays profiling reported overexpression of some -E2F1/2/3- in breast carcinomas [^31^ and References therein]. We quantified E2Fs expression in the RNA-Seq BRCA subtypes dataset. The box plots of Fig. S6A show the results of such analysis: most E2Fs have increased expression, particularly E2F1, E2F7 and E2F8. E2F4 was increased only in Basal-like and E2F6 was unchanged. E2F genes, with the exception of E2F1/2/4, have multiple isoforms, due to alternative promoters and/or alternative splicing events, but no data is available on their relative expression levels in BRCA. We analyzed them and found that isoforms predominantly expressed in normal tissues are also maintained in cancer cells (Fig. S6B). We conclude that there is indeed global overexpression of E2F genes in BRCA, notably the transcriptional “activating” members of the family, but not a switch in isoforms from normal to cancer cells.

### Switch of NF-YA isoforms in BRCA

**Figure 3:**
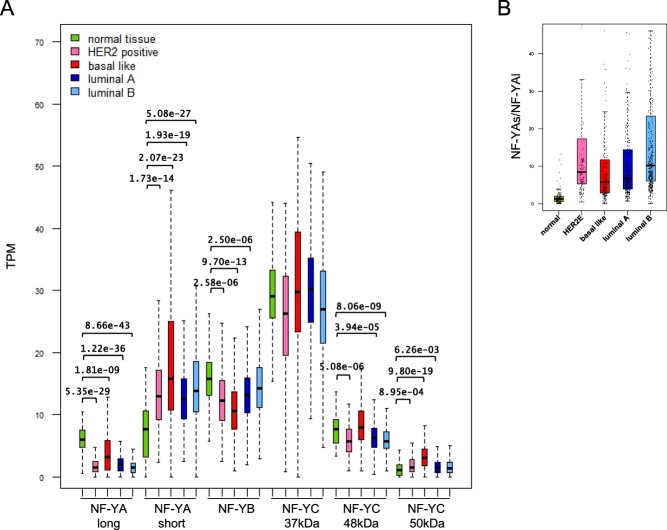
Analysis of expression of NF-YA isoforms in BRCA. (**A**) Expression of NF-Y subunits isoforms in the TCGA-BRCA, measured in TPM. The p-values are calculated using a Wilcoxon signal-ranked test and only values lower than 10^−3^ are shown. (**B**) Boxplots represent the ratio between NF-YAl and NF-YAs isoforms in the TCGA-BRCA cohort. The breast cancer samples are divided according to the PAM50 subtype.

NF-YA^5^ and NF-YC^6^ genes are subject to alternative splicing: the first originates two major isoforms, the latter multiple. We analyzed individual isoforms in BRCA subtypes. Figure 3A shows that in normal cells there are balanced levels of the two NF-YA isoforms; the “long” isoform -NF-YAl- was substantially decreased in HER2E, Luminal A and Luminal B, less so in Basal-like. The “short” -NF-YAs- was increased in all cohorts. As a result, a dramatic change in the ratios of the isoforms is produced, somewhat less striking in Basal-like tumors, mainly because of the relative higher levels of NF-YAl (Fig. 3B). As for NF-YC, there is a modest increase in the 48kD and 50kD isoforms, which likely accounts for the increase observed in the overall levels of this subunit (Fig. 2A); however, the 37kD isoform remains predominant, and largely invariant in tumors (Fig. 3A). We also controlled these events in the cohorts of 514 BRCA tumors previously classified by TCGA, and obtained very similar results (Fig. S7). We conclude that there is an isoforms switch in NF-YA, but not NF-YC, from normal to tumor cells.

### Expression of NF-YA isoforms in breast cancer cell lines

**Figure 4:**
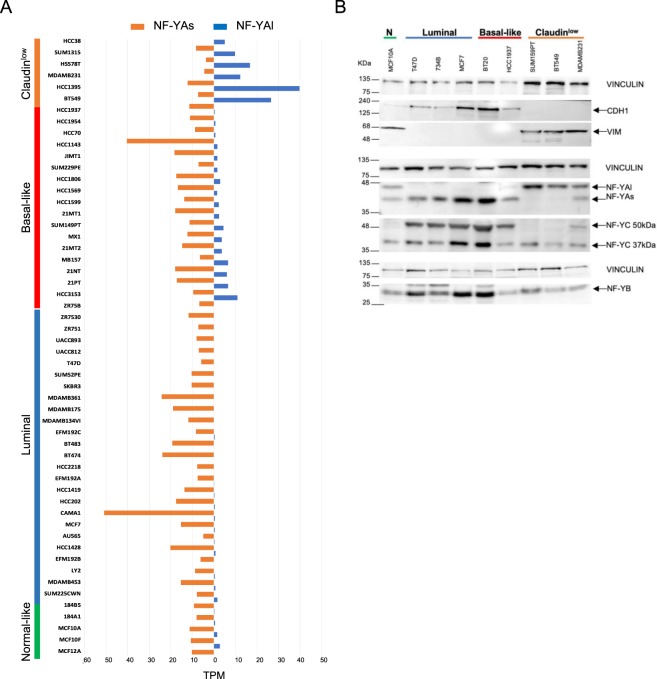
Analysis of mRNA and protein expression of NF-YA isoforms in 54 BRCA cell lines. (**A**) NF-YAs and NF-YAl isoforms expression in the 54 cell lines of the GSE48213 GEO dataset, measured in TPM. Cell lines are divided according to their subtype. (**B**) Western blot analysis of epithelial (CDH1), EMT (Vimentin) markers (Upper Panels), as well as NF-Y subunits (NF-YA and NF-YC, Middle Panels; NF-YB, Lower Panel) protein levels in the indicated representative breast cancer cell lines. N indicates the Normal-like MCF10A cells. Vinculin is used as an internal loading control for the different Panels. Full-length blots are shown in Fig. S8.

An independent verification of the NF-YA isoforms switch could be assessed in cell lines, partially recapitulating subtype specific features of breast cancers^32,33^. GEO data exists with RNA-Seq experiments of 54 BRCA cell lines (GSE48213)^34^. We analyzed the levels of NF-YA isoforms and the results are shown in Fig. 4A: in non-tumorigenic cells, NF-YAs predominates, and NF-YAl levels are low, but detectable. On the other hand, none of the Luminal lines have significant amounts of NF-YAl, which is also the case in Basal-like; however, those classified as Claudin^low^ do show much higher levels of NF-YAl, with a ratio higher, or close to 1. To substantiate the mRNA data, we examined by Western Blots extracts of selected cell lines (Fig. 4B). EMT markers were shown to be high only in Claudin^low^ cell lines^35^: we verified this at the protein level performing Western Blot analysis in SUM159PT, BT549, MDAMB231: these lines expressed Vimentin, unlike Luminal and Claudin^high^ Basal-like cells. In parallel, the epithelial marker CDH1 -E-Cadherin- showed the opposite pattern, not expressed in Claudin^low^ cells and high in the other cell lines. As for NF-YA, the three Luminal cells -T47D, 734B, MCF7- and two Basal-like -BT20, HCC1937- show almost exclusive expression of NF-YAs (41 kDa), as expected from the mRNA data. The normal-like MCF10A show similar amounts of NF-YAs and NF-YAl (45 kDa), with a NF-YAl/NF-YAs ratio close to 1. Importantly, the three Claudin^low^ cell lines have almost exclusively NF-YAl. Finally, we analyzed the expression levels of NF-YB and NF-YC. The NF-YC 37 kDa isoform was expressed, albeit not at equivalent levels, in all cells, whereas the 50 kDa isoform was not expressed -or less abundant- in Claudin^low^ cells (Fig. 4B). Note that, the NF-YA and NF-YC isoforms are in agreement with our previous observations that cells having NF-YAs have high levels of NF-YC 50 kDa, and those with NF-YAl have predominantly the 37 kDa^5^. As for NF-YB, it is expressed in all cells, although the levels are somewhat variant. In summary, mRNA and protein analysis of BRCA cell lines confirm the NF-YA isoform switch found in tumors, pointing to further molecular interrogation of BRCA tumors with low levels of Claudins, as to the relative levels of NF-YAl.

### Partitioning of BRCA tumors according to NF-YAl/NF-YAs ratios

**Figure 5:**
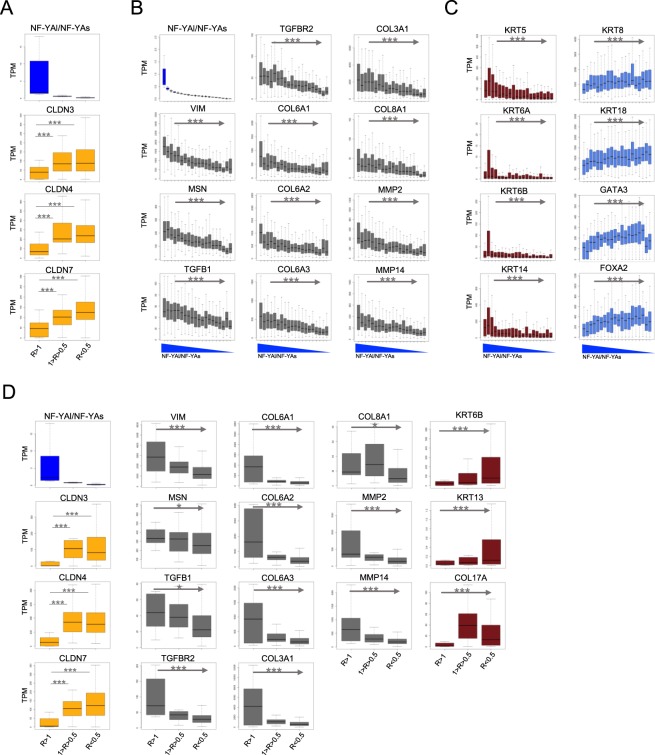
Analysis of TCGA tumors partitioned according NF-YAl/NF-Ys ratios. (**A**) Claudin 3/4/7 expression across TCGA-BRCA tumor samples grouped according to the ratios between NF-YAl and NF-YAs expression (R). The p-values are calculated using a Wilcoxon signal-ranked test. *p-value < 10^−3^, **p-value < 10^−4^, ***p-value < 10^−5^. (**B** and **C**) Analysis of expression across BRCA ranked samples of the indicated markers: EMT (grey), basal epithelia keratins (dark red) and luminal keratins (light blue). Ranking of BRCA tumor samples was based on ratios between NF-YAl and NF-YAs expression. Jonckheere-Terpstra trend test was used to assess significance in gene expression increasing or decreasing trend. *p-value < 10^−3^, **p-value < 10^−4^, ***p-value < 10^−5^. (**D**) Same as B, except that PAM50 Basal-like subtype tumor samples were considered for the analysis.

We analyzed the 1083 BRCA dataset according to the expression of Claudin3/4/7, considered as the relevant members of this family in BRCA^36^. First, we partitioned tumors in three subgroups having NF-YAl/NF-YAs ratios of R >1, R 1–0.5 and R < 0.5 (Fig. 5A, Upper Panel). We analyzed the levels of Claudin3/4/7 in the three cohorts (Fig. 5A Lower Panels): the box plots show that cohorts with high NF-YAl/NF-YAs ratios display the lowest Claudins levels.

EMT markers are also increased in Claudin^low^ cell lines (^35^; Fig. 4B). Therefore, we ranked all tumors according to NF-YAl/NF-Ys ratios, sampling them in 20 ordered groups (Fig. 5B, Upper Panel) and analyzed 11 EMT markers: all show a significantly decreasing trend from high to low NF-YAl/NF-YAs ratios. In parallel, the levels of basal epithelial markers -Keratin 5/6A/6B/14- show a decrease, as for EMT genes (Fig. 5C, left Panels). Nevertheless, the very first cohort, corresponding to NF-YAl^high^ Claudin^low^ presumably Basal-like tumors, displays low expression of basal Keratins. Their levels are substantially higher in the second cohort, likely also Basal-like, but with high levels of Claudins and lower ratios of NF-YAl/NF-YAs. Finally, consistent with previous analysis^35^, Keratin 8/18, not expressed in basal epithelia, show an opposite pattern of expression (Fig. 5C, Right Panels): low in Basal-like, progressively higher in the tumors with very low NF-YAl/NF-YAs ratios: these presumably correspond to Luminal subtypes, in which Keratin 8/18 are known to be expressed. To substantiate this point, we analyzed GATA3 and FoxA1, two TFs overexpressed in Luminal subtypes (^28^ and References therein): Fig. 5C (Right Panels) shows indeed an expression pattern very similar to Keratins 8/18.

It is largely assumed that most of the Claudin^low^ tumors are Basal-like. We therefore repeated the same analysis on the 203 tumors classified as such (Fig. S3). In the three subgroups divided according to isoforms ratios, Claudin3/4/7 expression was indeed found to be lowest in the cohort with highest NF-YAl/NF-YAs ratio (5D, Upper Panel). EMT markers globally behaved in the opposite way, being low in those with low ratios (5D, Right Panels). These data confirm that the NF-YAl^high^ Claudins^low^ population is part of the Basal-like.

Altogether, we conclude that increased expression of the NF-YA “long” isoform marks a specific cohort of Basal-like tumors with low levels of Claudins, of basal epithelial Keratins, and high levels of markers typical of Epithelial to Mesenchymal Transition.

### Correlation of NF-YAl/NF-YAs ratios with clinical outcomes of BRCA

**Figure 6:**
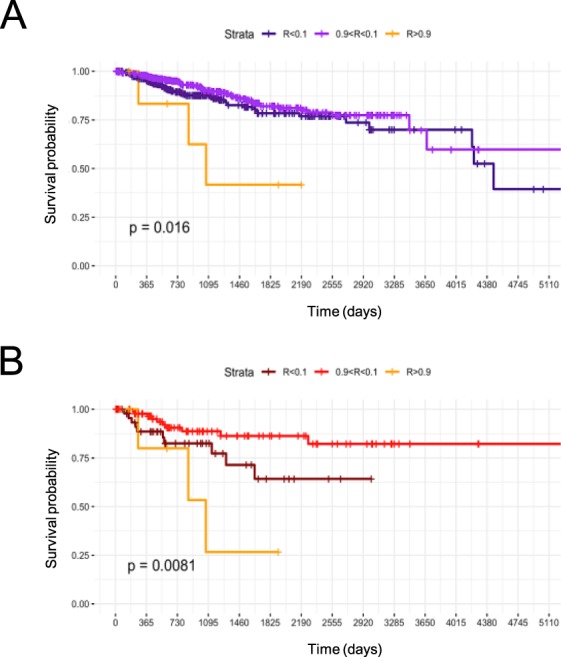
Prognostic value of NF-YAl/NF-YAs ratios in breast cancer. (**A**) Kaplan–Meier plots of survival probability of patients stratified by NF-YAl/NF-YAs ratio (R > *0*.*9*, *0*.*1* < *R* < *0*.*9*, *R* < *0*.*1*). (**B**) Kaplan–Meier plots of survival probability of Basal-like tumors stratified by NF-YAl/NF-YAs ratio (R > *0*.*9*, *0*.*1* < *R* < *0*.*9*, *R* < *0*.*1*). p-values were calculated by log rank test.

For a substantial number of BRCA samples -758- clinical data of patients are available, specifically clinical outcomes, such as Progression Free Intervals (PFI). We stratified all available tumors in the three NF-YAl/NF-YAs ratios: >0,9, >0,1 <0,9 and <0,1. The Meyer-Kaplan curves of Fig. 6A show a significant drop (p value 0,016) in the -small- cluster of patients with isoforms ratios >0,9, that is, with a prevalence of NF-YAl.

The bulk of the patients, however, were not significantly affected by lower ratios. This might result from the fundamentally different outlook of LuminalA/B vs Basal-like tumors. In addition, 489 Luminal A/B are present, compared to 149 Basal-like. We therefore, repeated the analysis taking into consideration only Basal-like: Fig. 6B shows that the relative few patients described above with a high NF-YAl/NF-YAs ratio and short PFI are all in this subtype of tumors. In addition, it also shows that patients with the opposite spectrum of isoforms -very low NF-YAl/NF-YAs ratio, hence NF-YAs predominating- do show a shorter PFI. This is not as dramatic as in those with high NF-YAl/NF-YAs ratio, but still significant (p value 0,0081).

Altogether, these data are in agreement with the presence of tumors with a severe unbalance of NF-Y isoforms having a prognosis worst than those having intermediate levels of the two isoforms. These cohorts are exclusively among Basal-like tumors.

## Discussion

### The NF-Y trimer in cancer cells

Abundance of CCAAT boxes was repeatedly reported in promoters of genes overexpressed in cancer, but not in down-regulated genes, including in the data presented here. Such genes contain “proliferative” signatures, enriched in cell-cycle regulatory genes, often with terms “*mitosis*” or “*G2/M*”: NF-Y sites are notoriously essential for activation of this class, specifically G2/M promoters^7^. In addition, the importance of metabolic changes in tumor cells brought re-evaluation of the expression levels of “housekeeping” metabolic genes, long time considered more or less invariant: NF-Y location and functional analysis found rate-limiting genes leading to changes in cancer cell metabolism as NF-Y targets^36^. The data presented here (Fig. 2) are strong evidence that this rewiring is a generalized phenomenon, at least in BRCA. We identified 840 genes commonly overexpressed in the four subtypes, corresponding to the pro-proliferative signatures illustrated in past analysis of 514 and 817 tumors^26,27^. These genes contain CCAAT in promoters and belong to GO terms typical of NF-Y targets. Taken together, these data indicate that NF-Y is pivotal in the activation of “cancer” genes in BRCA, and quite possibly in other epithelial cancers.

NF-YA is thought to be the regulatory, potentially rate-limiting subunit of the TF trimer. All three subunits, and thus the CCAAT-binding activity, are inevitably present in immortalized or transformed cells growing in culture, but some non-transformed cells, such as terminally differentiated circulating monocytes and post-mitotic myotubes lack, or have little NF-YA^37,38^. The data depict a scenario whereby NF-YA overexpression in tumors will engage with an excess HFD dimer to increase functional trimer formation; in turn, this would *de novo* activate genes switched off in normal cells, or sustain high expression of CCAAT-dependent growth-promoting genes. The question is now whether NF-YA mRNA levels are increased due to promoter-mediated activation, post-transcriptional mechanisms, or both. As for the latter mechanism, at least one lncRNA -PANDAR- has been implicated in NF-YA mRNA regulation^39^. A third level of control regards NF-YA protein half-life, which is generally short -1-2 hours- and controlled by post-translational modifications^40^. A comparison between mRNA and protein levels in BRCA cell lines suggests good concordance, but this issue will have to be further verified in cancer specimens. While not all types of tumors have elevated levels of NF-YA, as exemplified here by analysis of GBM, it is obvious that epithelial tumors -lung, colon, stomach, liver, among others- will have to be further investigated in quantitative and qualitative terms.

### NF-YA isoforms in breast cancer

The role of the two major NF-YA splicing isoforms has been obscure for decades. Structurally, they differ in 28/29 amino acids located in the Q- and hydrophobics-rich Trans-Activation Domain (TAD), thus sharing the same HFD-interaction and DNA-binding domain. As a consequence, they both trimerize and bind DNA with apparent identical affinities. Experiments in two non-transformed systems -Hematopoietic Stem cells and mouse Embryonic Stem cells- indicate that NF-YAs is more abundant in “stem”, NF-YAl in differentiated cells^41,42^. We find that NF-YAl is high, although not predominant, in normal breast tissues (Fig. 4): since there are different types of epithelial and myoepithelial cells, it is impossible to determine the cell-specific expression. The normal-like MCF10A have high levels of NF-YAs transcript, yet the protein level of NF-YAl is at least as abundant as NF-YAs, suggesting important post-transcriptional/translational control of expression.

**Figure 7:**
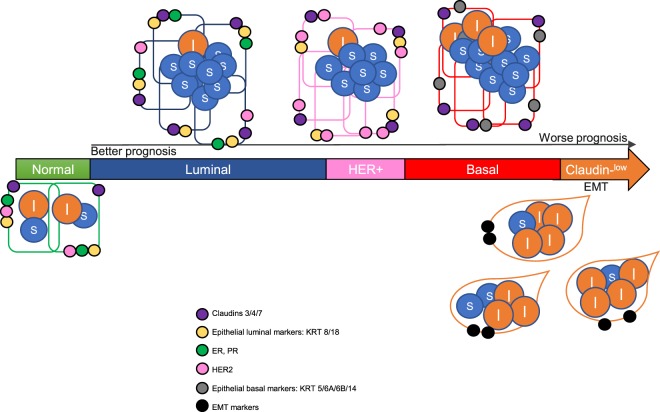
Representative scheme of NF-YA isoforms partitioning in normal and breast cancer. The different types of BRCA are shown, with progressively worst prognosis from left to right. Circles indicate the relative levels of NF-YAl (l, orange) and NF-YAs (s, blue), Claudin3/4/7 (purple), HER2 (pink), ER and PR (green), Basal markers (grey), Luminal markers (yellow) and EMT markers (black).

We took advantage of the PAM50 signature classifier to complete the partitioning of all TCGA BRCA tumors in the four canonical subtypes. In Luminal A, Luminal B and HER2E tumors, NF-YAs is high, very low levels of NF-YAl are scored (Fig. 7). The same is observed in BRCA cell lines catalogued as Luminal, where NF-YAl mRNA is often below detectable limits and virtually no NF-YAl protein is revealed in Western blots. We were certainly not prepared to observe the remarkable NF-YA mRNA differences among the different Luminal lines, up to 10-fold between CAMA1 and AU565. It will be important to determine whether this systematically translates into variation of protein levels.

On the other hand, the picture is different in Basal-like tumors, which correspond, to a large extent, to the Triple Negative -ER^−^/PR^−^/ERBB2^−^-classification: both NF-YAs and NF-YAl are increased (Fig. 7). This is not widespread in all tumors, since there is clear partitioning in two distinct subgroups, with NF-YAl^high^ and NF-YAl^low^ mRNA levels. In essence, most BRCA tumors are Claudin^high^ and NF-YAl^low^. Claudin^low^ tumors are more aggressive, pharmacologically challenging, prone to metastasize and have a worse prognosis. Further analysis of clinical data confirms the worst Progression-Free-Interval (PFI) curves in tumors with a high NF-YAl/NF-YAs ratio, that are NF-YAl^high^ ones. Driven by our analysis of Claudin^low^ cell lines, we found a population of NF-YAl^high^ and Claudin^low^ tumours. This explains the higher NF-YAl/NF-YAs ratio in Basal-like, compared to the other subtypes (Fig. 3). Claudin^low^ NF-YAl^high^ tumors have also high levels of the 11 markers analyzed as hallmarks of EMT, as well as the expected low levels of basal Keratins. Among the EMT genes analyzed here, only TGFBR2 is a *bona fide* NF-Y target, as determined in breast cancer cells^43^. Note that NF-Y function is apparently complex, since it could be repressive of TGFBR2 promoter function under certain conditions^44^. There is ground to think that the specific isoform present in the cellular context and/or stimulus might have a role in this regulation. None of the other 10 EMT genes analyzed has CCAAT in promoters, nor NF-Y-binding is scored *in vivo*. This suggests that the activity of NF-Y on these EMT genes is indirect. ZEBs, Twists, SNAIs, are all TFs involved in EMT, identified as highly expressed in Claudin^low^ cells^35^: we remark that SNAI1 and SNAI2, but not the other TFs, have canonical CCAAT in promoters, bound by NF-Y *in vivo*. We are tempted to speculate that higher levels of NF-YAl in Claudin^low^ tumors are involved in programs of increased expression of mesenchymal genes through the activity of these TFs. A differential role of NF-YAl vs NF-YAs on these EMT mediators is worth testing. At the other hand of the spectrum, Claudin^high^ Basal-like tumors with very high levels of NF-YAs, and negligible ones of NF-YAl, show shorter PFIs, although not at the same level, suggesting that an overall unbalance of the NF-YA isoforms predict an aggressive behavior, most likely through different molecular mechanisms.

Our finding about the importance of the NF-YAl/NF-YAs ratios might have practical fallouts: by measuring this parameter, which is doable by qRT-PCR, one could predict a mesenchymal phenotype of the specific BRCA tumor, and therefore a more aggressive behavior, irrespective upon molecular assessment of other epithelial or mesenchymal markers. Appropriate clinical analysis will be required to prove this point. Along this line, it would be desirable to have an antibody recognizing the 28/29 aminoacids coded by Exon 3, hence NF-YAl specifically, for this would prove valuable for IHC studies.

### Partnership between NF-Y and E2Fs

The genes activated in all four subtypes of BRCA constituting the “proliferative signature” have NF-Y, E2Fs and GC-rich sequences enriched in their promoters. The GC-rich sequences are most likely bound and activated by members of the vast Zn-finger SP/KLF family of TFs. The NF-Y connection with this family, particularly Sp1/2, is well established (Reviewed in^45^). The link with E2Fs to activate proliferative, cell cycle and metabolic genes is also robust, based on dissection of individual promoters and on ChIP-Seq data of ENCODE and independent studies, showing E2F1 and E2F4 frequently binding close to NF-Y/CCAAT *in vivo*^1,46,47^). Many E2Fs genes were found overexpressed in profiling experiments of different types of cancers, including BRCA [^32^ and References therein]. We extend these observations to the whole set of BRCA RNA-Seq data in TCGA. As for the various isoforms of E2F genes, none of them shows a significant switch in transformed cells. In general, a more abundant NF-Y trimer and specific E2F member might cooperate in activation of the aforementioned group of promoters. The genomic analysis performed so far has not revealed any obvious positional preference between the binding sites^1,47^; nevertheless, it is reasonable to think that a better understanding of the NF-Y/E2Fs interplay at the structural level will shed some light on the coordinate overexpression patterns observed.

### No role of HFD subunits in cancer

The mRNA levels of the HFD subunits are relatively high in cancers, but not with consistent up- or down-regulation across the majority of TCGA tumors. In selected tumors, either NF-YB and/or NF-YC are overexpressed, but the data would tend to suggest that the HFDs are generally passive players in CCAAT-dependent activation of cancer signature genes. However, two recent studies indicate the opposite, that they are “driver” oncogenes. (i) Follicular lymphomas often evolve aggressively in Diffuse Large B-cell Lymphoma (DLBCL): in tumors carrying the frequent translocation involving BCL2, RNA profilings identified NF-YB among few TFs playing a crucial role^48^. (ii) Mouse/human genomic screenings searching for driver oncogenes of Choroid Plexus Carcinomas identified the syntenic NF-YC, TAF12 and RAD54L genes, with the formers having the greatest impact on tumor development^49^. In this respect, the GBM data are intriguing, since none of the NF-Y subunits are increased at the mRNA levels, yet the levels of the NF-YC protein were reported increased in these tumors^22^. In addition, we were somewhat surprised by the relatively unequal levels of NF-YB protein, specifically, in the BRCA cell lines analyzed. In summary, before ruling out a role of HFDs in tumors, we should consider the possibility that HFDs are mostly regulated at a translational or post-translational, rather than transcriptional level. Further analysis with appropriate techniques is necessary to verify this hypothesis.

## Methods

### Bioinformatic analysis

TCGA gene and isoforms data of primary tumors and normal samples were retrieved from http://firebrowse.org/ webpage as RSEM preprocessed Tables. For the Gene Expression Omnibus projects (https://www.ncbi.nlm.nih.gov/geo/), fast-q files were retrieved using the fastq-dump utility of the SRA-toolkit 2.3.2 version. mRNA expression was analyzed using RSEM-1.17 with the default parameters for paired-end data.

For differential expression, we used the DESeq2 package of Bioconductor. Deregulated genes were selected using the following conditions: Log2 fold change >2/<−2 and FDR <0.01 for upregulated and downregulated genes. For pathway enrichment analysis, we used KOBAS 3.0 Gene-list enrichment web server (http://kobas.cbi.pku.edu.cn/anno_iden.php). For over-represented Transcription Factor Binding Site motifs analysis we used Pscan^29^, selecting a promoter region of −450 +50 nucleotides from the TSS and using the Jaspar 2018_NR descriptor. For *de novo* motif discovery, promoter sequences (−450 +50 nucleotides from the TSS) of deregulated genes were retrieved from UCSC Genome Browser and used as input of the Weeder tool^30^.

For breast cancer samples classification, we used the *genefu* R package^50^, and PAM50 algorithms, in a multi-tiered compendium of bioinformatics algorithms and gene signatures for molecular subtyping and prognostication in breast cancer. R suite was used to create all the graphics, boxplots and statistics. In particular, we evaluated statistical relevance between groups of expression values using Wilcoxon signed-rank test. Jonckheere-Terpstra trend test was applied to assess differences in gene expression across *a priori* ordered conditions.

Survival analyses were performed on survival Progression Free Interval (PFI) of TCGA BRCA data, downloaded from https://nborcherding.shinyapps.io/TRGAted/. Estimated probability of overall survival was calculated with the Kaplan-Meier method using R package *survminer*. Survival curves statistics were performed with Log-rank (Mantel-Cox) Test.

### Cell lines and western blot analysis

Human breast cell lines were derived from ATCC and kindly donated by Dr. N. Zaffaroni (Istituto Nazionale dei Tumori, Milano) and cultured under standard ATCC conditions. MCF10A were grown in DMEM/F12 supplemented with insulin (5 μg/ml), hydrocortisone (1 μg/ml), EGF (20 ng/ml) and cholera toxin (100 ng/ml). MDAMB231, T47D, 734B were grown in DMEM/F12 supplemented with 10% Fetal Bovine Serum (FBS). MCF7 and BT549 were grown in RPMI supplemented with 10% FBS and insulin (5 μg/ml). BT20 and SUM159PT were grown in DMEM/F12 supplemented with 10% FBS and insulin (5 μg/ml). HCC1937 were grown in RPMI supplemented with 10% FBS, non-essential aminoacids (1%) sodium pyruvate (1%). All cell lines media were supplemented with 1 mM L-glutamine, 100 μg/mL penicillin, 100 μg/ml streptomycin. Whole cell extracts and Western blots were performed according to standard procedures, with anti-NF-YA G2 (Santa Cruz Biotech., sc-17753), anti-Vinculin (Sigma-Aldrich 05-386), anti-NF-YB and anti NF-YC (home made), anti-CDH1 (Santa Cruz Biotech., sc-8426), anti-Vimentin (Santa Cruz Biotech., sc-6260) primary antibodies and a peroxidase conjugated secondary antibody (Sigma-Aldrich). Detection was performed with ChemiDoc (Bio-Rad) and exported using Image-Lab software (Bio-Rad).

## Supplementary information

Supplementary Figures

Supplementary Table 1

Supplementary Table 2

Supplementary Table 3

## Data Availability

All data generated or analyzed during this study are included either in this article or in the Supplementary Information Files.
