# Supplementary material for: Overexpression and alternative splicing of NF-YA in breast cancer
Source: Sci Rep. 2019 Sep 10;9:12955. doi: 10.1038/s41598-019-49297-5 (PMC6736888; doi:10.1038/s41598-019-49297-5)
Supplement: Supplementary file 1 — Supplementary Figures [file 41598_2019_49297_MOESM1_ESM.pdf]

Overexpression and alternative splicing of NF-YA in breast cancer.

Diletta Dolfini[1], Valentina Andrioletti[1°] and Roberto Mantovani[1*].

[1] Dipartimento di Bioscienze, Università degli Studi di Milano, Via Celoria 26, 20133 Milano, Italy.

° Current Address: Internal Medicine VIII, University Hospital Tübingen. Otfried-Müller-Str. 14, 72076 Tübingen, Germany

*To whom correspondence should be addressed.

Current e-mail addresses:

Diletta Dolfini, diletta.dolfini@unimi.it

Valentina Andrioletti, valentina.andrioletti@med.uni-tuebingen.de

Roberto Mantovani, mantor@unimi.it;

Tel. 39-02-50315005

Supplementary Figure 1.
Expression of NF-YA, NF-YB and NF-YC subunits, expressed in FPKM (Fragments Per Kilobase Million), as retrieved from the Firebrowse portal. On the x axis there are the acronym of the TCGA projects. Green boxes represent expression in the normal tissue samples, red boxes represent the tumor samples. On the top of each figure there are the p-values resulted from the Wilcoxon signal-ranked test between the distribution of the normal samples expression and the primary tumor expression. Red stars signal significant overexpression (p-value $<10^{-4}$), while green stars highlight significant down-regulation. The table lists the acronyms of the acronyms of the TCGA projects and the number of normal tissues and tumors.

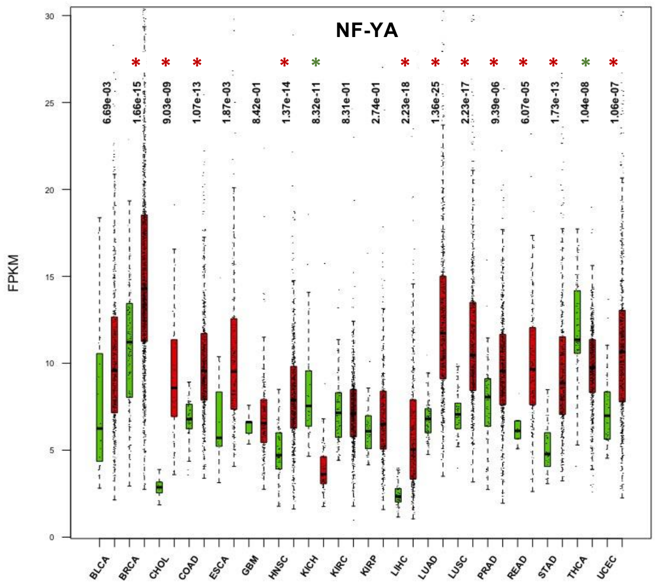

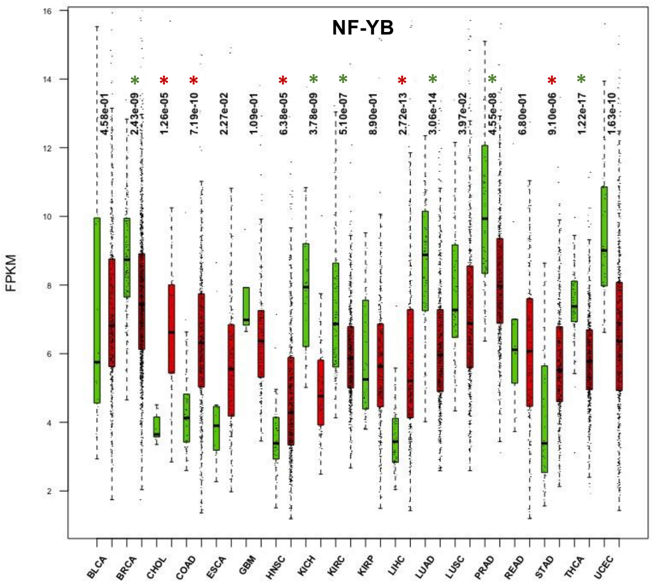

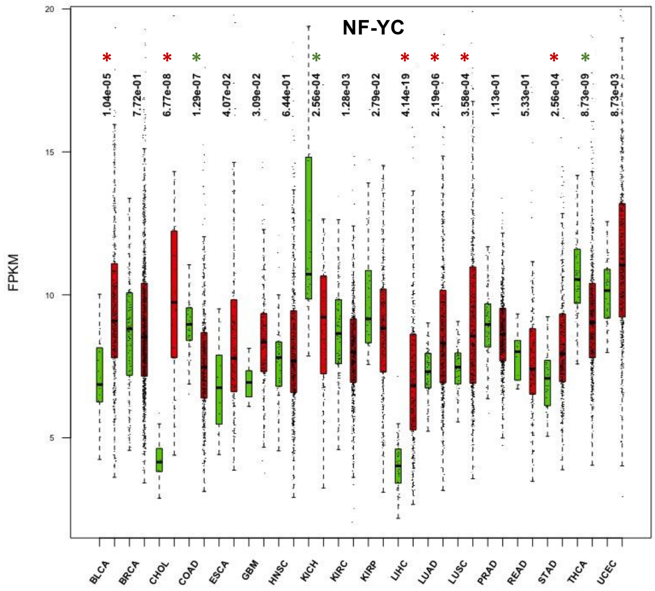

	NORMAL	TUMOR
BLCA	19	414
BRCA	113	1102
CHOL	9	36
COAD	41	478
ESCA	11	161
GBM	5	156
HNSC	44	500
KICH	24	65
KIRC	72	538
KIRP	32	288
LIHC	50	371
LUAD	59	533
LUSC	49	502
PRAD	52	498
READ	10	166
STAD	32	375
THCA	58	502
UCEC	35	551

Supplementary Figure 2.
Box plots represent the expression of NF-Y subunits at gene level in the GBM GEO dataset GSE59612, measured as reads counts. The p-values are calculated using a Wilcoxon signal-rank test.

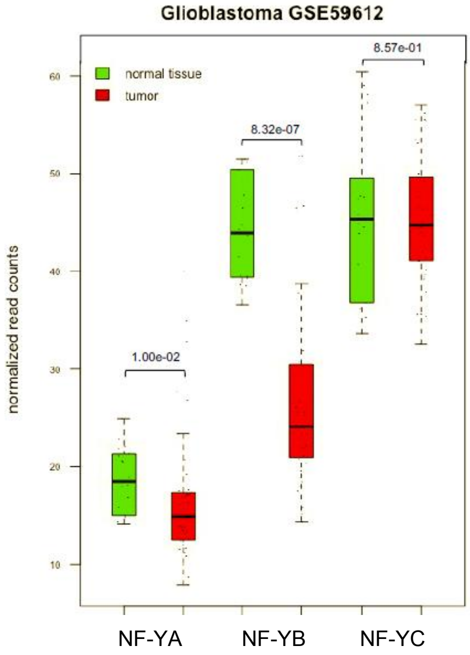

Supplementary Figure 3.

Venn diagrams represent the overlap between the PAM50 classification published by TCGA [26] in blue circles, in Ciriello et al. [27] in black circles, and our classification in red circles. The number of cases is indicated in the correspondent area.

Luminal A

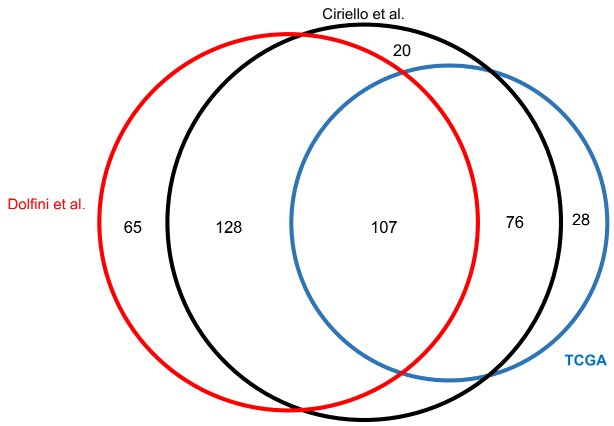

Luminal B

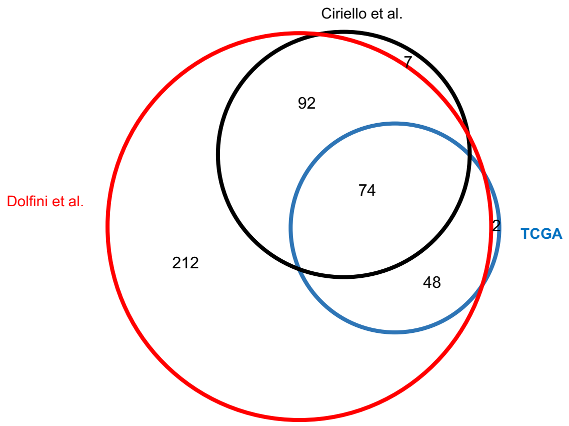

HER2E

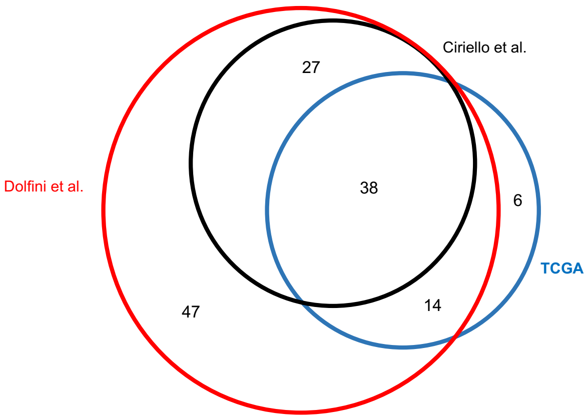

Basal-like

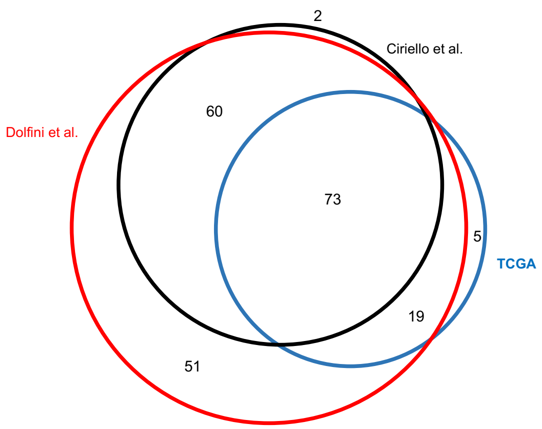

Supplementary Figure 4.
Heatmap showing expression of the PAM50 genes signature across the TCGA breast cancer samples. Gene symbols are listed on the right. In order to obtain a centered key color, the log2 transformation of raw read counts was used. Clustering of samples was made with the centroid option.

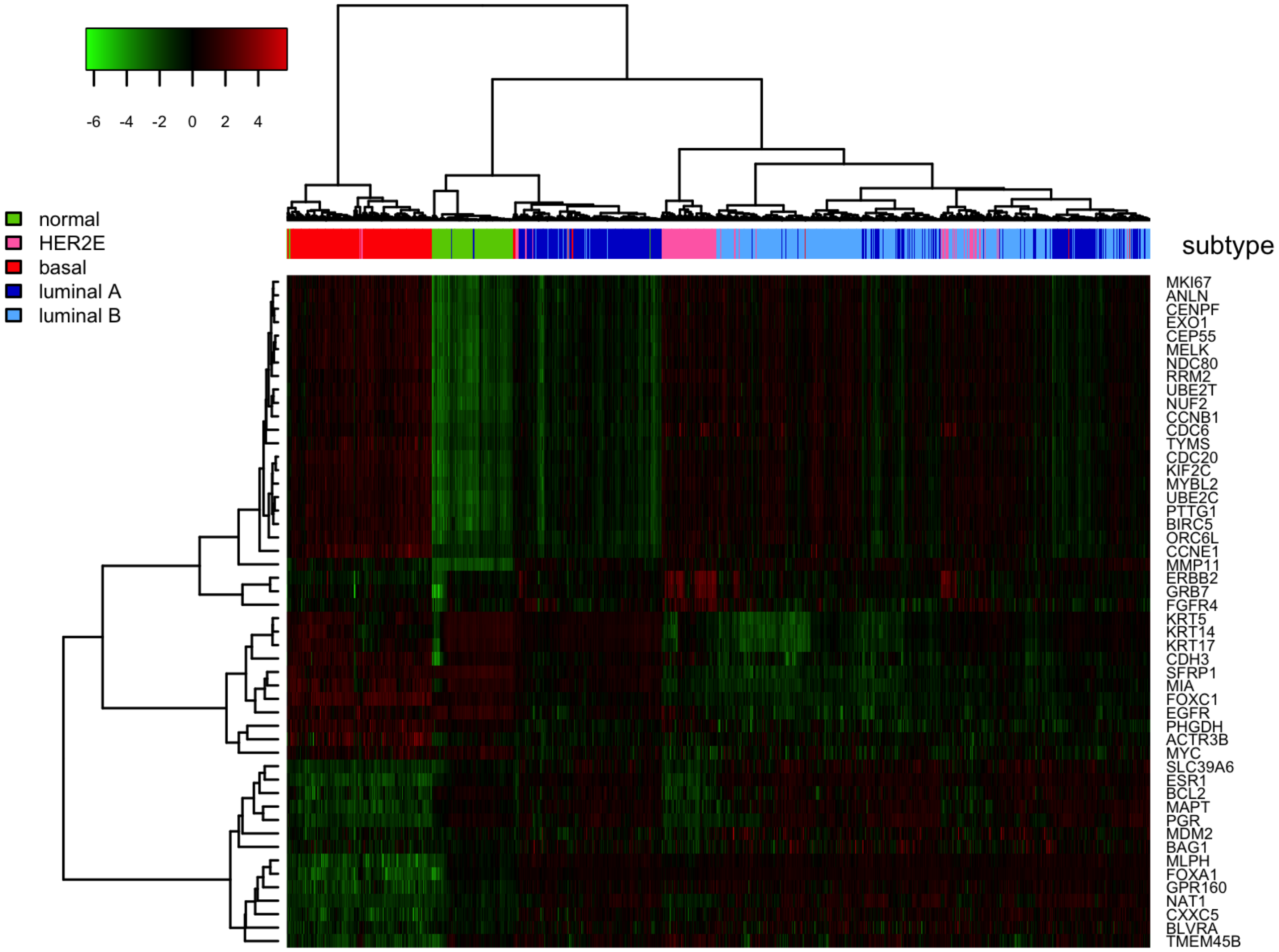

Supplementary Figure 5.
Venn diagrams represent the downregulated genes for each PAM50 subtype, compared to normal tissue, in TCGA-BRCA: in the center, the number of genes commonly downregulated in all 4 subtypes; on the borders, the genes exclusively downregulated in each group. For subtype-specific and common downregulated genes, the most represented TFBSs enriched in promoters using Pscan are listed.

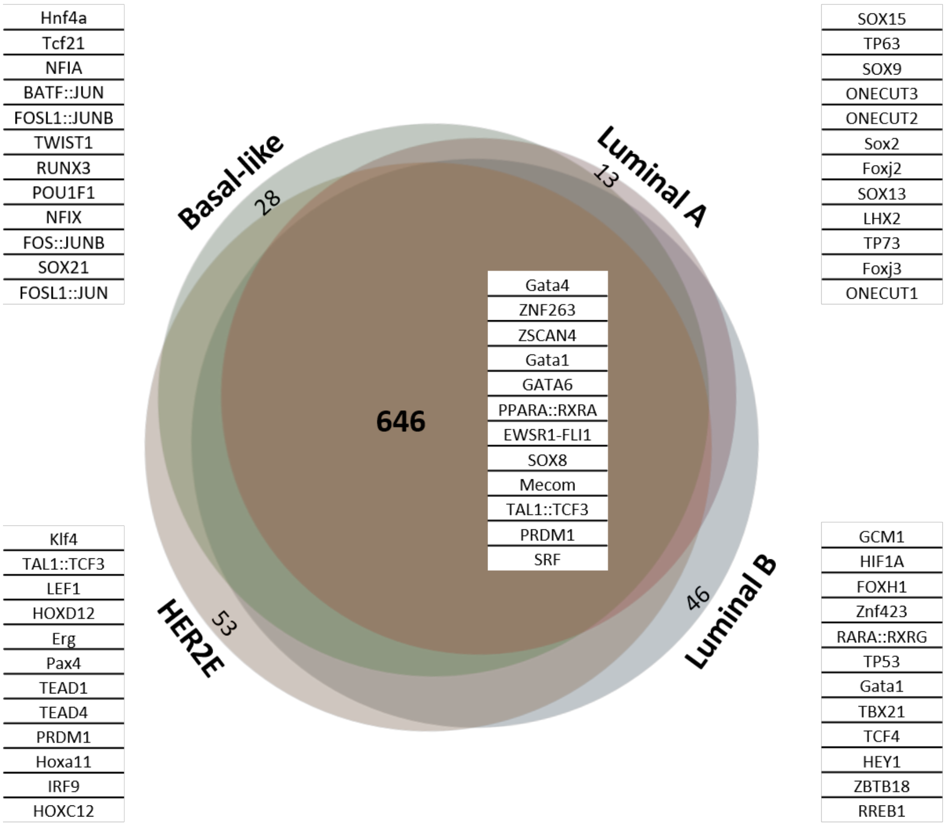

Supplementary Figure 6.
A. Expression of the E2F family members at gene level across TCGA-BRCA, measured as TPM. The breast cancer samples are divided according to the PAM50 subtypes. p-values are calculated using a Wilcoxon signed-rank test and only values lower than 10^{-3} are shown. **B.** Box plots show the expression of E2F family TFs at isoforms level in TCGA-BRCA, measured in TPM (Transcripts Per Million). The breast cancer samples are divided according to the PAM50 subtypes. The p-values are calculated using a Wilcoxon test and only the values lower than 10^{-3} is shown.

A

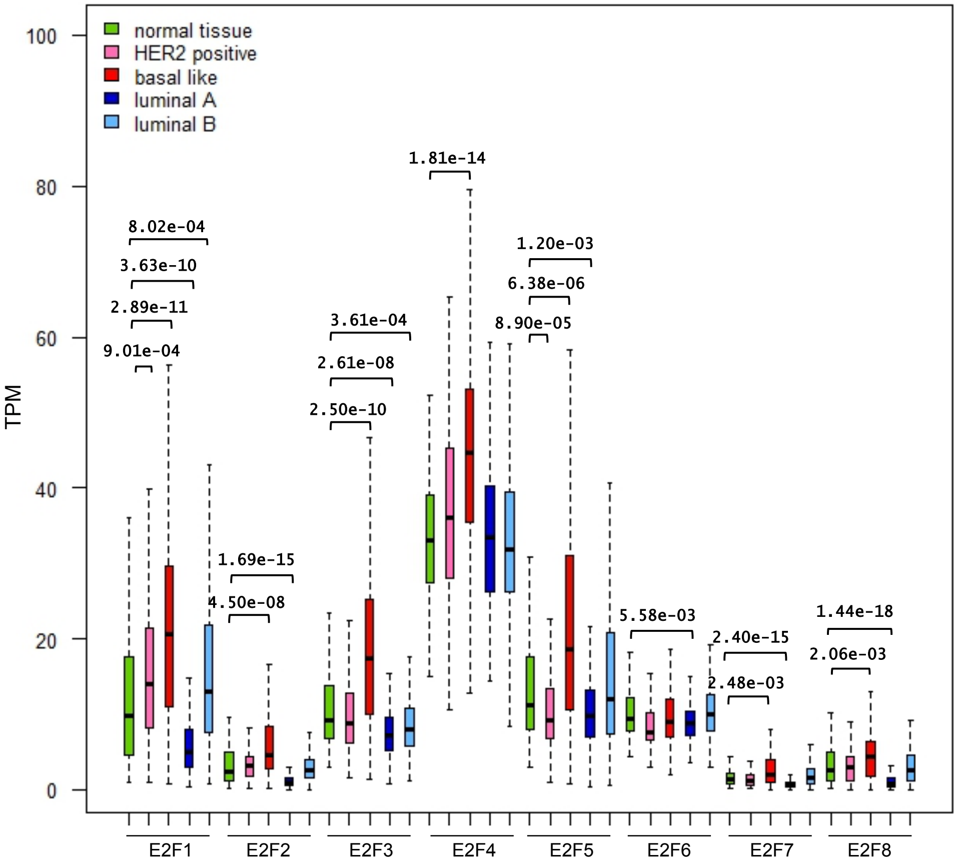

B

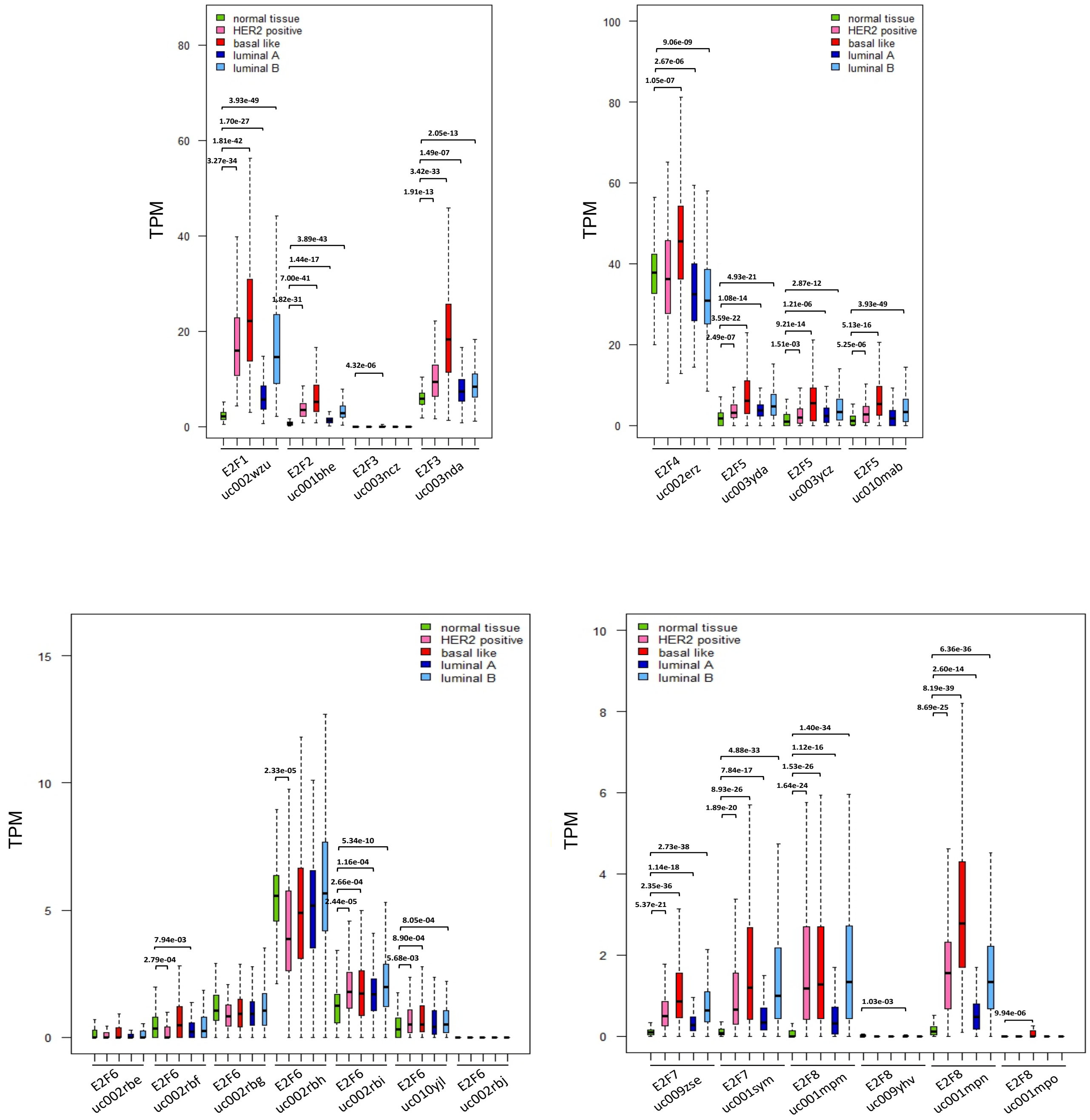
normal tissue
HER2 positive
basal like
luminal A
luminal B
TPM
3.93e-49
1.70e-27
1.81e-42
3.27e-34
3.89e-43
1.44e-17
7.00e-41
1.82e-31
4.32e-06
2.05e-13
1.49e-07
3.42e-33
1.91e-13
E2F1 uc002wzu
E2F2 uc001bhe
E2F3 uc003ncz
E2F3 uc003nda
9.06e-09
2.67e-06
1.05e-07
4.93e-21
1.08e-14
3.59e-22
2.49e-07
2.87e-12
1.21e-06
9.21e-14
1.51e-03
3.93e-49
5.13e-16
5.25e-06
E2F4 uc002erz
E2F5 uc003yda
E2F5 uc003ycz
E2F5 uc010mab
2.33e-05
7.94e-03
2.79e-04
5.34e-10
1.16e-04
2.66e-04
2.44e-05
8.05e-04
8.90e-04
5.68e-03
E2F6 uc002rbe
E2F6 uc002rbf
E2F6 uc002rbg
E2F6 uc002rbh
E2F6 uc002rbi
E2F6 uc010yjl
E2F6 uc002rbj
2.73e-38
1.14e-18
2.35e-36
5.37e-21
4.88e-33
7.84e-17
8.93e-26
1.89e-20
1.40e-34
1.12e-16
1.53e-26
1.64e-24
1.03e-03
6.36e-36
2.60e-14
8.19e-39
8.69e-25
9.94e-06
E2F7 uc009zse
E2F7 uc001sym
E2F8 uc001mpm
E2F8 uc009yhv
E2F8 uc001mpn
E2F8 uc001mpo

Supplementary Figure 7.
Box plots show expression of NF-Y subunits at isoforms level in TCGA-BRCA cohorts based on 514 tumor samples [26], measured in TPM (Transcripts Per Million). The breast cancer samples are divided according to the PAM50 subtype as in the indicated publication. The p-values are calculated using a Wilcoxon signal-rank test and only values lower than 10^{-3} are shown.

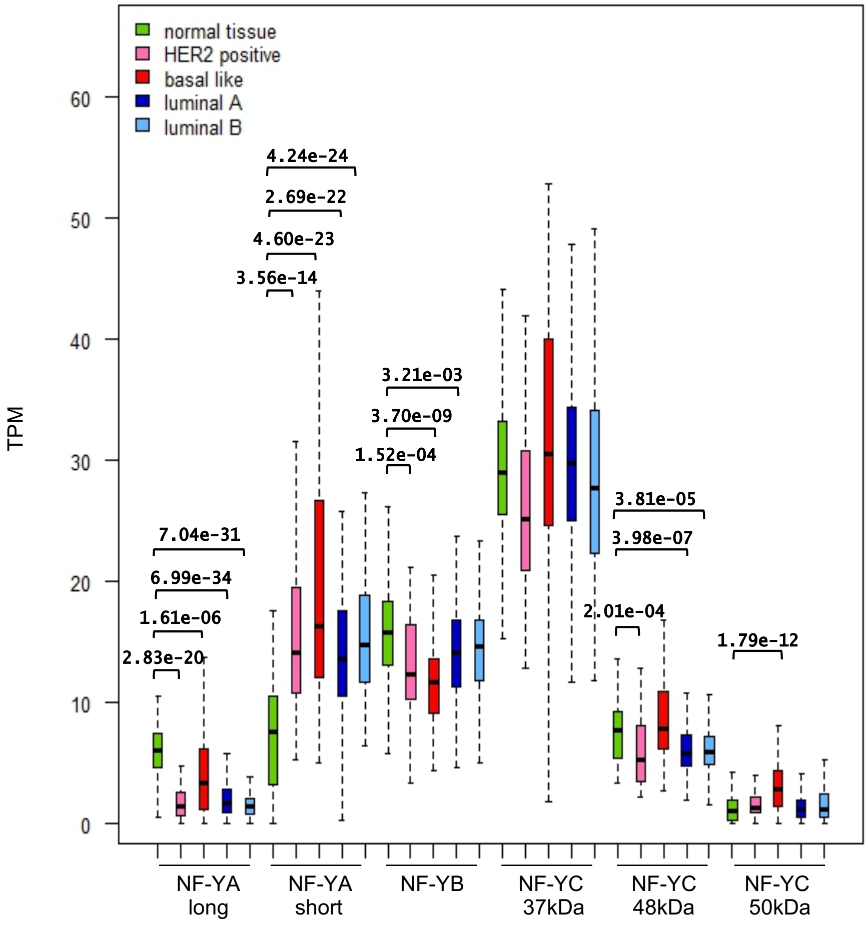

Supplementary Figure 8
Full-length western blots images of Figure 4

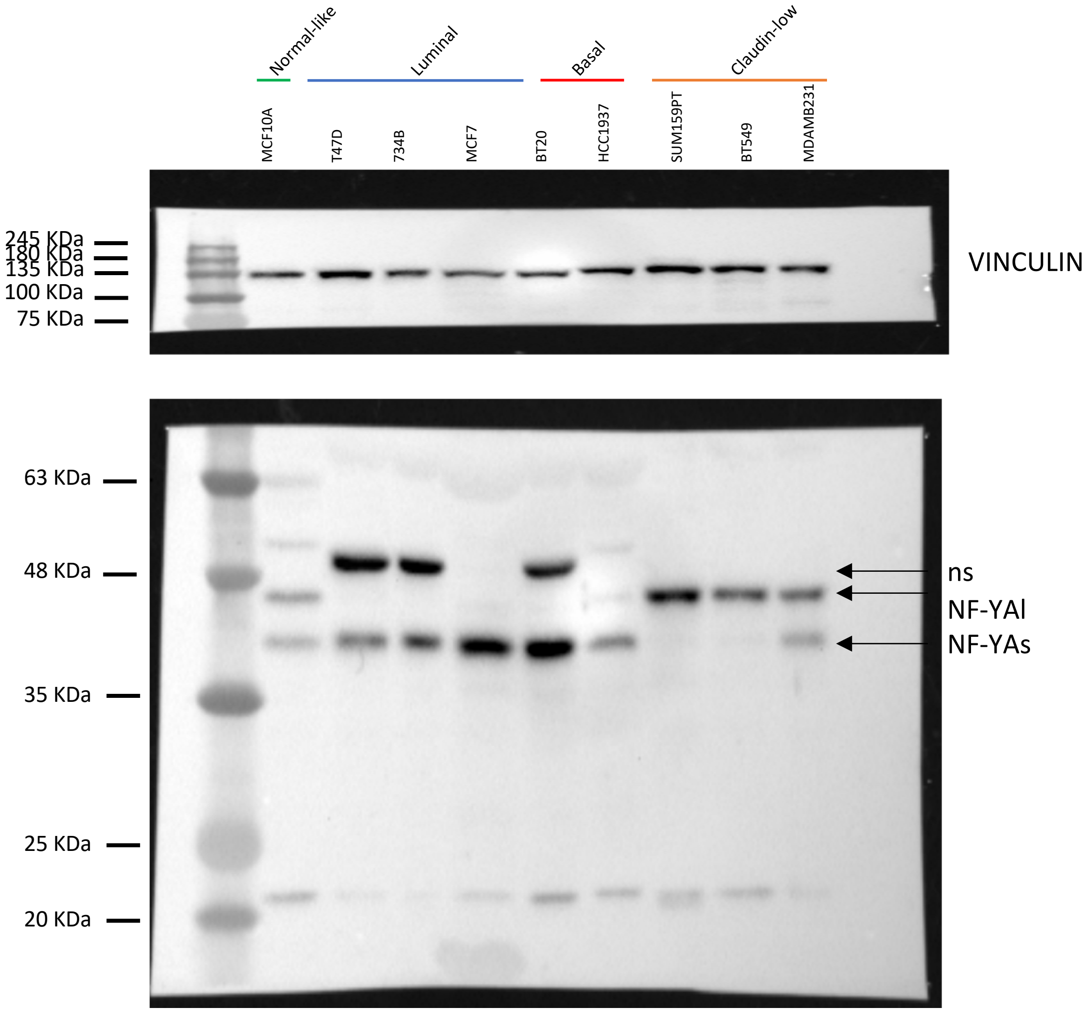

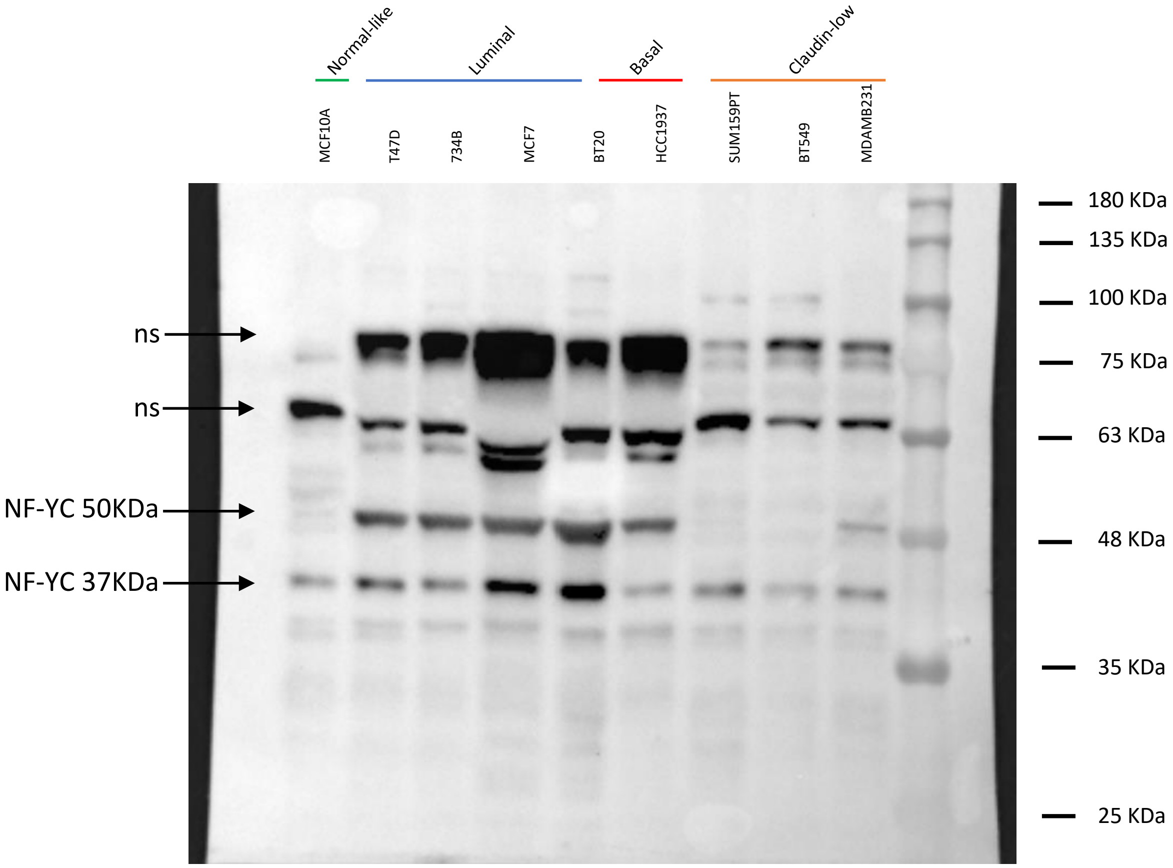
Normal-like
Luminal
Basal
Claudin-low
MCF10A
T47D
734B
MCF7
BT20
HCC1937
SUM159PT
BT549
MDAMB231
ns
ns
NF-YC 50KDa
NF-YC 37KDa
180 KDa
135 KDa
100 KDa
75 KDa
63 KDa
48 KDa
35 KDa
25 KDa

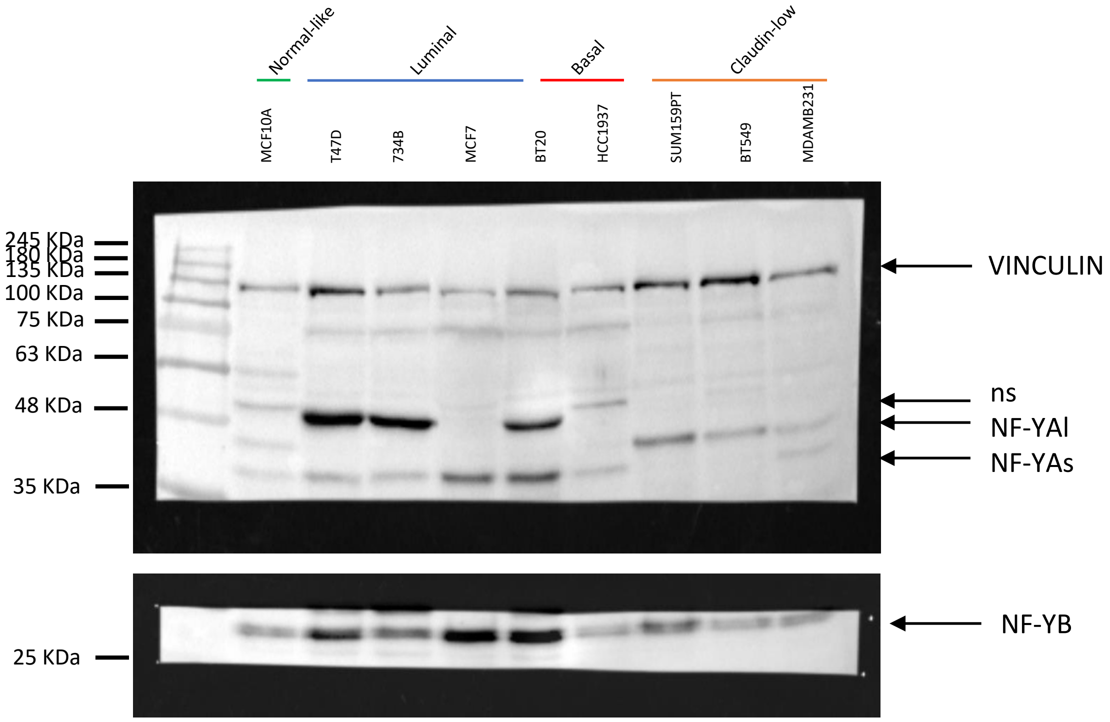
Normal-like
Luminal
Basal
Claudin-low
MCF10A
T47D
734B
MCF7
BT20
HCC1937
SUM159PT
BT549
MDAMB231
245 KDa
180 KDa
135 KDa
100 KDa
75 KDa
63 KDa
48 KDa
35 KDa
VINCULIN
ns
NF-YAl
NF-YAs
25 KDa
NF-YB

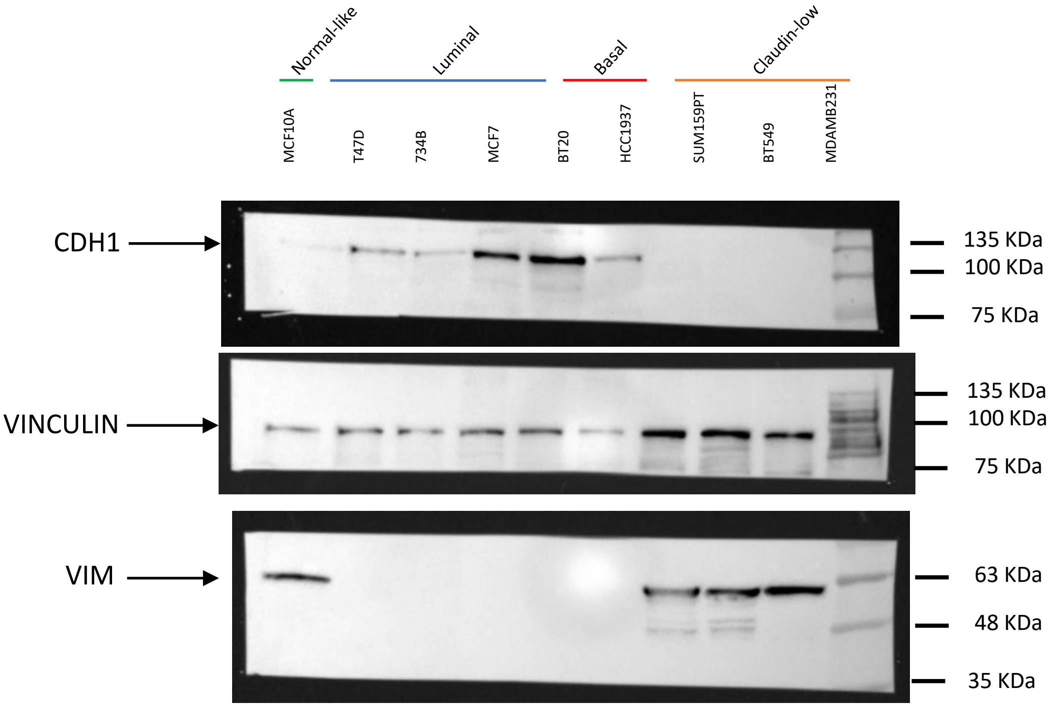

Normal-like
Luminal
Basal
Claudin-low
MCF10A
T47D
734B
MCF7
BT20
HCC1937
SUM159PT
BT549
MDAMB231
CDH1
VINCULIN
VIM
135 KDa
100 KDa
75 KDa
135 KDa
100 KDa
75 KDa
63 KDa
48 KDa
35 KDa

Overexpression and alternative splicing of NF-YA in breast cancer.
Diletta Dolfini, Valentina Andrioletti and Roberto Mantovani.

Supplementary Table 1.
List of the 1083 breast carcinomas, classified according to the PAM50-derived four subtypes.

Supplementary Table 2.
Differential mRNA expression analysis based on TCGA BRCA RNA-seq data.

Supplementary Table 3.
Lists of overexpressed and underexpressed genes in BRCA subtypes. Commonly and subtype-specific signatures are classified.
